# Correction: Hydrogen-activation mechanism of [Fe] hydrogenase revealed by multi-scale modeling

**DOI:** 10.1039/d1sc90165f

**Published:** 2021-08-09

**Authors:** Arndt Robert Finkelmann, Hans Martin Senn, Markus Reiher

**Affiliations:** Laboratory of Physical Chemistry, ETH Zürich Vladimir-Prelog-Weg 2 Zürich Switzerland markus.reiher@phys.chem.ethz.ch; WestCHEM and School of Chemistry, University of Glasgow Glasgow G12 8QQ UK hans.senn@glasgow.ac.uk

## Abstract

Correction for ‘Hydrogen-activation mechanism of [Fe] hydrogenase revealed by multi-scale modeling’ by Arndt Robert Finkelmann *et al.*, *Chem. Sci.*, 2014, **5**, 4474–4482, DOI: 10.1039/C4SC01605J.

The authors regret that there were minor typographical errors in two figures. In Fig. 9 and 11, the internuclear distances were swapped. The Fe-bound hydrogen atoms are affected, where H_p_ is the hydrogen atom proximal to the oxypyridine ligand and H_d_ is the hydrogen atom distal to the oxypyridine ligand. In Fig. 9, left panel, the distance between H_p_ and the oxypyridine O atom was given as 1.82 Å and the distance between H_p_ and the Fe atom was given as 1.7 Å. However, it should read 1.82 Å between H_p_ and Fe and 1.70 Å between H_p_ and the oxypyridine O atom. In Fig. 11, top left panel, the distance between H_p_ and Fe was shown to be 1.70 Å and the distance between H_d_ and Fe was given as 1.73 Å. However, it should read 1.73 Å between H_p_ and Fe and 1.70 Å between H_d_ and Fe. The correct versions of these figures are given below. The results and conclusions are not affected by these typographical errors.

**Fig. 9:**
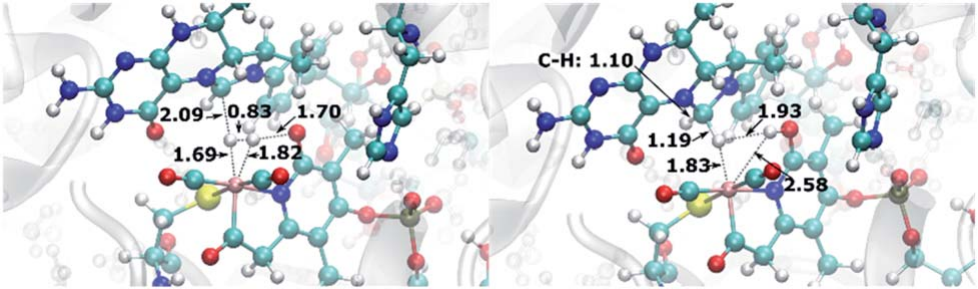
QM/MM-optimized reactant (left) and product (right) structures of the H_2_ cleavage reaction for the scenario with oxypyridine ligand. Distances are given in Å.

**Fig. 11:**
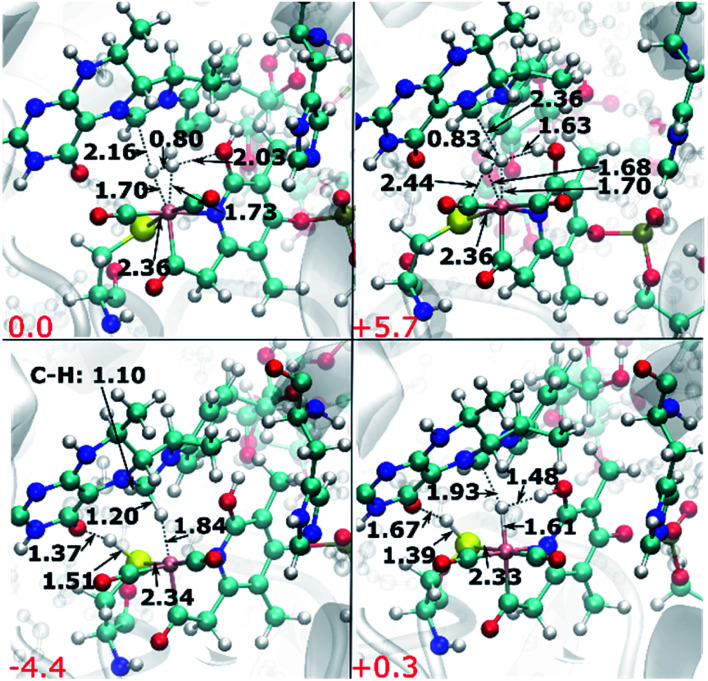
Top row: structures of the H_2_ adduct for the second scenario with neutral pyridinol; the pyridinol OH can be oriented away from Fe (top left) or towards Fe (top right). Bottom row: products of H_2_ cleavage, with the proton transferred to the thiolate; with the hydroxyl oriented away from Fe (bottom left) and towards Fe (bottom right). Distances are given in Å; relative energies with respect to the favoured adduct are indicated in red in kcal mol^−1^.

The Royal Society of Chemistry apologises for these errors and any consequent inconvenience to authors and readers.

## Supplementary Material

